# Cannabis and Cannabinoids in the Treatment of Rheumatic Diseases

**DOI:** 10.5041/RMMJ.10389

**Published:** 2020-01-30

**Authors:** Tal Gonen, Howard Amital

**Affiliations:** 1 Sackler Faculty of Medicine, Tel-Aviv University, Tel-Aviv, Israel; 2 Department of Medicine ‘B’ & The Zabludowicz Center for Autoimmune Diseases, Sheba Medical Center, Tel-Hashomer, Ramat-Gan, Israel

**Keywords:** Cannabinoids, cannabis, chronic pain, fibromyalgia, rheumatic diseases

## Abstract

Chronic pain is a common complaint among patients, and rheumatic diseases are a common cause for chronic pain. Current pharmacological interventions for chronic pain are not always useful or safe enough for long-term use. Cannabis and cannabinoids are currently being studied due to their potential as analgesics. In this review we will discuss current literature regarding cannabinoids and cannabis as treatment for rheumatic diseases. Fibromyalgia is a prevalent rheumatic disease that causes diffuse pain, fatigue, and sleep disturbances. Treatment of this syndrome is symptomatic, and it has been suggested that cannabis and cannabinoids could potentially alleviate some of the symptoms associated with fibromyalgia. In this review we cite some of the evidence that supports this claim. However, data on long-term efficacy and safety of cannabinoid and cannabis use are still lacking. Cannabinoids and cannabis are commonly investigated as analgesic agents, but in recent years more evidence has accumulated on their potential immune-modulatory effect, supported by results in animal models of certain rheumatic diseases. While results that demonstrate the same effect in humans are still lacking, cannabinoids and cannabis remain potential drugs to alleviate the pain associated with rheumatic diseases, as they were shown to be safe and to cause limited adverse effects.

## INTRODUCTION

Chronic pain is commonly defined as pain that lasts for longer than three to six months and is a common complaint among many patients seeking medical attention.^1^ The prevalence of chronic pain among the adult population in certain countries is estimated to be as high as 30%,^2^ and rheumatic diseases are a leading cause for chronic pain.^3^ Analgesia in rheumatic diseases is often an important part of treatment, especially since disease remission and response to therapy do not always entirely eliminate pain. In rheumatoid arthritis (RA) patients, it had been shown that pain can persist even with the achievement of clinical targets, and that pain was also the most common residual symptom associated with RA remission or low disease activity.^4^ In this review, we will discuss the potential of using cannabis and cannabinoids in the treatment of rheumatic disease, based on the literature existing on this issue.

Management of chronic pain is difficult, and patients are often unsatisfied with the effect of treatment.^5^ Drug options that are currently available may not be very safe for certain patient populations. Opioids are a problematic long-term solution for chronic pain, due to the risk they carry of significant adverse events, addiction, and overdose.^6^ Opioid use was also found to be associated with more severe symptoms and unemployment in fibromyalgia.^7^ Other drugs used to treat chronic pain, such as antidepressants (e.g. serotonin-norepinephrine reuptake inhibitors [SNRIs], tricyclic antidepressants [TCAs]), have been shown to be useful for this indication but have certain side effects (e.g. increased risk of cardiovascular events and falls with TCAs) that might limit their use in older patients.^8^ One solution for long-term pain that has been studied in the context of pain relief in rheumatic diseases—but not thoroughly enough—is the use of cannabis or cannabinoids, which may potentially show therapeutic qualities as well.^9^

## CANNABINOIDS

It is assumed that the plant *Cannabis sativa* exerts its effects on human physiology through substances it contains, termed phytocannabinoids (over 100 of them have already been isolated so far). Those phytocannabinoids are thought to bind cannabinoid receptors throughout the human body, to which endocannabinoid (i.e. cannabinoids produced by human tissue) bind as well. Of the phytocannabinoids, tetrahydrocannabinol (THC) and cannabidiol (CBD) are the most well-studied and are used as medications. Tetrahydrocannabinol is considered to be the more psychoactive component in cannabis, while CBD is considered to be the major non-psychoactive component. Cannabinoid receptors are found in a variety of tissues throughout the body—from neurons in the frontal cortex, to the gastrointestinal tract and immune cells as well.^9^ According to the “entourage theory,” the combination of THC and CBD creates a synergistic effect in which other phytocannabinoids possibly take part as well, suggesting that there could be a benefit in using cannabis rather than synthetic cannabinoids as analgesic or therapeutic agents.^10^

## FIBROMYALGIA

Fibromyalgia is a common chronic pain syndrome causing diffuse pain, tenderness, fatigue, and sleep disturbances. Other complaints include cognitive symptoms, as well as headaches.^11^ The prevalence of fibromyalgia is estimated at 2.7% globally.^12^ Without a known pathophysiology and etiology, and therefore in the absence of disease-modifying or definitive treatment, analgesia is a significant part of fibromyalgia symptomatic treatment. Fibromyalgia patients may respond to certain pharmacological agents (e.g. antidepressants and anticonvulsants) or to other interventions such as aerobic exercise, physical therapy, and rehabilitation programs (non-pharmacological interventions were recommended as the first line of treatment in recent European League Against Rheumatism [EULAR] guidelines^13^).

Fibromyalgia pain shares certain common characteristics with neuropathic pain,^14^ and both are thought to involve a mechanism of central sensitization.^15^ It should also be noted that current guidelines recommend treating it with similar agents to those used in neuropathic pain.^13^

Ibuprofen, a non-steroidal anti-inflammatory drug (NSAID) usually used for the treatment of musculoskeletal pain, was not found to be an effective treatment option,^16^ and a randomized double-blinded study that compared the addition of etoricoxib, a selective COX-2 inhibitor, to pre-existing medical therapy with the addition of placebo in female fibromyalgia patients found that etoricoxib did not improve patients’ pain, sleep, or disability parameters.^17^ While tramadol (a weak opioid with mild SNRI activity) was found to be potentially effective in alleviating fibromyalgia pain,^13^ opioids in general may cause an exacerbation of symptoms in this patient population.^7^ Cannabis and cannabinoids were recommended for the treatment of neuropathic pain,^18^ and, due to the similarities between neuropathic pain and fibromyalgia, as previously mentioned, it is not unreasonable to hypothesize that cannabis or cannabinoids might be effective for fibromyalgia-associated pain as well.

Data regarding the use of cannabinoids in the treatment of fibromyalgia consist of several studies investigating the use of nabilone—a synthetic analog of THC—and fewer in which cannabis was used. Two studies evaluating the use of nabilone in fibromyalgia were included in a Cochrane review that found that nabilone was not superior to placebo or amitriptyline (a TCA) in relieving fibromyalgia symptoms,^19^–^21^ as neither study provided high/moderate-quality evidence for efficacy. However, one study included in this Cochrane review did show very low-quality evidence that nabilone compared with placebo led to a decrease in pain and anxiety as well as to an improvement in health-related quality of life.^21^ In the other study included in this Cochrane review, very low-quality evidence that nabilone was superior to amitriptyline in improving sleep was found.^20^ While cannabinoids were not suggested as treatment for fibromyalgia in the aforementioned Cochrane review, The National Academies of Science, Engineering, and Medicine suggested in their 2017 report that there was moderate evidence that cannabis or cannabinoids are effective for fibromyalgia.^22^

In an observative study in which 28 fibromyalgia patients treated with cannabis were compared with 28 controls, significant pain relief, reduction of stiffness, and increase in relaxation and perception of well-being were all found, and were evaluated by visual analog scale (VAS) before and 2 hours after cannabis self-administration.^23^ More compelling results emerge from a study that included fibromyalgia patients in Israel. In a recent publication by Sagy et al.,^24^ a prospective observational study was conducted, in which 367 fibromyalgia patients were treated with medical cannabis and followed up at six months. A total of 81.1% of patients achieved treatment response, and pain intensity decreased significantly from a median of 9 at baseline to 5 at six months (on a numeric rating scale of 0 to 10, with 0 being no pain, and 10 being worst pain imaginable). Dizziness, dry mouth, and gastrointestinal symptoms were among the most common side effects of the treatment. In a recent retrospective review, Habib and Artul^25^ assessed 26 fibromyalgia patients treated with medical cannabis, using the Fibromyalgia Impact Questionnaire. The mean duration of cannabis treatment was 10.4 months, and the mean dose of cannabis was 26 g per month. Significant improvement was reported in every item of the questionnaire after cannabis treatment, and 50% of patients stopped using any other medical therapy for fibromyalgia. Adverse effects were mild and were reported by 30% of patients.

In another study, Habib and Avisar employed questionnaires on social media to reach out to Israeli fibromyalgia patients using cannabis^26^ and found that, of 383 responders, 323 (84%) reported consuming cannabis; 142 (44%) of these were licensed to do so. The majority of patients reported pain relief (94%) and improved sleep quality (93%). Depression and anxiety were both also reported to improve under cannabis use by the patients. Most of the reported adverse effects were mild (e.g. eye or throat irritation); 12% reported experiencing adverse effects.

In another recent study that assessed the analgesic effect of inhaled cannabis with varying concentrations of THC and CBD, pressure and electrical pain thresholds, spontaneous pain scores, and drug high were measured before and after cannabis inhalation. The results showed that cannabis strains containing THC led to a significant increase in pressure pain threshold compared with placebo. However, no strain of cannabis was found to be superior to placebo’s effect on spontaneous or electrical pain responses. Drug high was assessed by the Bowdle questionnaire and was found to occur in 40%–80% of the subjects treated with inhaled cannabis, compared to 10% of the subjects in the placebo group.^27^

## RHEUMATOID ARTHRITIS, OSTEOARTHRITIS, AND SYSTEMIC SCLEROSIS

Cannabis and cannabinoids were investigated as substances that can ameliorate chronic pain and other symptoms associated with rheumatic disease. However, it had also been suggested that cannabinoids have an inflammatory-modulating quality that could exert a therapeutic effect in such conditions, as cannabinoids were shown to have an overall anti-inflammatory effect on immune cells; these results were reinforced by studies in animal models of RA and systemic sclerosis (SSc).^9^

In RA and osteoarthritis (OA), for example, the hypothesis that cannabinoids may have a disease-modifying quality is based on animal models, as well as *in vitro* studies that have shown that the synovia of RA and OA patients contained two endocannabinoids that the synovia of healthy controls did not. Results of the same study showed that, in fibroblast-like cells obtained from RA and OA patients, cellular receptors ERK-1 and ERK-2 underwent phosphorylation in response to cannabinoid stimulation, an effect which was attenuated by a cannabinoid receptor antagonist.^28^

In another study, synovial tissue obtained from RA patients was shown to undergo attenuation and inhibition of cytokine production in response to a cannabinoid binding a cannabinoid receptor.^29^ Animal models also suggest a possible therapeutic quality for cannabinoids in RA, with three studies using a murine model with collagen-induced arthritis showing a beneficial effect of the cannabinoids CBD, JWH-133, and HU-308. These substances were found to be associated with clinical improvement: CBD was associated with a decrease in cytokine release and production as well as a decrease in lymphocyte proliferation^30^; JWH-133 was associated with a decrease in serum antibody levels, decreased cytokine production, and reduced bone destruction^31^; and HU-308 was associated with less joint swelling and destruction, reduced synovial inflammation, along with a decrease in serum antibody levels.^32^ Despite these promising results, clinical research focusing on cannabinoids’ disease-modifying qualities is still lacking.

The use of cannabinoids for the relief of pain associated with RA has been assessed by one study^33^ which showed that, in comparison with placebo, the cannabis-based drug was associated with significant improvements in certain pain parameters and quality of sleep. With regard to drug safety, the study found no serious adverse effects in the active treatment group, with most adverse effects being mild or moderate.

In OA, a murine model with surgically induced OA showed that the severity of the disease was reduced in wild-type compared with mice that have undergone gene-deletion for a presumed relevant cannabinoid receptor. The same study also showed that treatment of wild-type mice with an agonist for the same cannabinoid receptor resulted in a partial protection against OA that did not occur in the gene-deletion group or in the wild-type placebo group.^34^ In another study, the activity of an enzyme suspected of causing cartilage breakdown was reduced by the treatment of chondrocytes from OA patients with a cannabinoid.^35^ Only one clinical trial assessed the use of an endocannabinoid modulator in OA for pain relief, and this was not found to be significantly more beneficial for OA-associated pain than placebo.^36^ Other clinical trials assessing the use of cannabis and cannabinoids for OA are currently ongoing or are yet to be published.^37^

Several studies have also shown that cannabinoids and cannabinoid receptors might play a role in SSc, as cannabinoid receptors have been shown to modulate SSc in murine models,^9^ and were also found to be over-expressed in SSc fibroblasts.^38^ A study on a murine model also found that treatment with cannabinoids prevented the development of cutaneous and pulmonary fibrosis and decreased the proliferation of fibroblasts and antibody development.^39^ A clinical trial of a novel oral selective cannabinoid receptor agonist is currently in phase 3, after showing a statistically significant effect on skin fibrosis.^40^

Research from recent years has shown some promising results regarding the potential of cannabinoids as disease-modifying therapeutics in rheumatic disease. To further investigate this theory, clinical trials should be conducted to evaluate the disease-modifying quality of cannabis in certain rheumatic diseases.

However, despite the evidence on the potential of cannabis and cannabinoids in the treatment of rheumatic disease and the pain associated with it, the literature regarding the use of cannabis as treatment for chronic pain in general contains conflicting reports. While The National Academies of Science, Engineering, and Medicine found in their 2017 report that there was substantial evidence that cannabis or cannabinoids effectively managed chronic pain in adults,^22^ and in 2015 an updated review of randomized controlled trials suggested that cannabinoids are a reasonable treatment option for chronic non-cancer pain, being safe and “modestly effective,”^41^ other reviews were less supportive of those claims.

An overview of systematic reviews on the efficacy and safety of cannabis-based medications for chronic pain concluded that there was insufficient information to recommend cannabinoids as treatment for chronic pain in rheumatic disease,^42^ and a systematic review and meta-analysis from 2018 on the treatment of non-cancer chronic pain with cannabis and cannabinoids claimed that the number needed to treat to benefit was high and the number needed to treat to harm was low, and that the evidence for effectiveness of cannabinoids for chronic non-cancer pain was insufficient.^43^ It should be emphasized that while the reviews cited in this paragraph evaluated studies and systematic reviews in which cannabis was used to treat chronic pain of many etiologies, in this article we wish to focus on the potential of cannabis as treatment for chronic pain caused by rheumatic diseases only. In a recent review of this topic, Sarzi-Puttini et al.^44^ discussed the pros and cons of medical cannabis in the treatment of rheumatic diseases, claiming that, given the evidence currently available, cannabis should only be used as complementary treatment in rheumatic diseases at the moment, until high-quality evidence is found.

## CONCLUSION

In conclusion, we believe that the use of cannabis and cannabinoids for pain relief in rheumatic diseases (and fibromyalgia in particular) shows great potential and may be a source of hope for those suffering from chronic pain associated with those conditions, and for the physicians treating them. More research into this question should be conducted, especially among larger cohorts of patients and for longer periods of time, to assess for long-term efficacy and adverse effects.^45^ At this point, the data suggest that the use of cannabinoids and cannabis carries limited side effects in the treatment of rheumatic disease,^46^ although drug interactions should always be kept in mind.^9^ Research also suggests that cannabis and cannabinoids can improve some common and debilitating symptoms of rheumatic disease, thus making them an adequate potential treatment option in our opinion, when other treatment lines have been exhausted.

## Abbreviations

CBD: cannabidiol
EULAR: European League Against Rheumatism
NSAID: non-steroidal anti-inflammatory drug
RA: rheumatoid arthritis
SNRI: serotonin-norepinephrine reuptake inhibitor
SSc: systemic sclerosis
OA: osteoarthritis
TCA: tricyclic antidepressant
THC: tetrahydrocannabinol
VAS: visual analogue scale.
